# Mucin in benign thyroid nodules: treatment related change or not?

**DOI:** 10.1186/s13044-019-0062-4

**Published:** 2019-01-11

**Authors:** Adriana Handra-Luca

**Affiliations:** 1 Service d’Anatomie pathologique, APHP GHU Avicenne, Universite Paris Nord Sorbonne Cite, 125 rue Stalingrad, 93009 Bobigny, France; 2 Universite Paris Nord, UFR SMBH, Bobigny, France

**Keywords:** Thyroid, Benign nodule, Mucin, Treatment

**Fig. 1:**
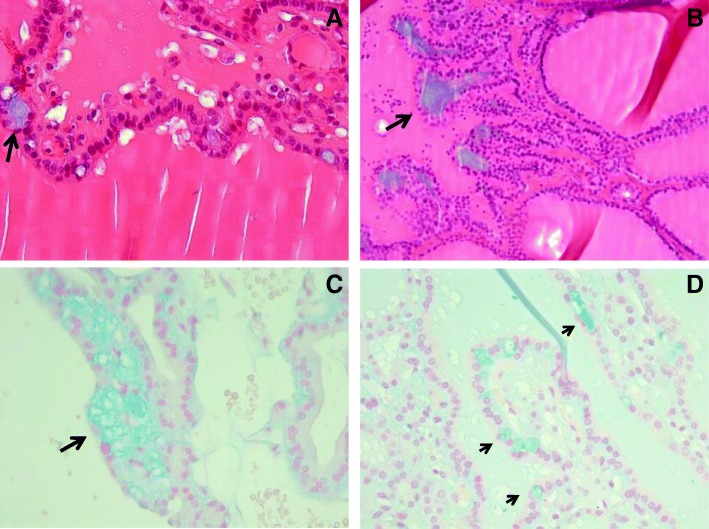
The thyroid nodules showed subepithelial, stromal mucin (**a**-**d**: black arrows), stained blue on Alcian blue stain. Hematoxilin and eosin stain **a**, **b**; Alcian blue stain **c**, **d**. Original magnification × 20 A, × 10 B × 40 (**c**, **d**)

To the Editor,

Sir,

We have read with great interest the report by Namulema et al. [1] of increased gastric mucin after thyroxin administration in indomethacin (1-(4-chlorobenzoyl)-5-méthoxy-2-méthyl-1-H-indole-3-acetic acid)-induced ulcer healing in Wistar rats. We had the opportunity to observe extracellular mucin in 3 thyroidectomy specimens (resections for toxic goiter). Mucin is rare in the thyroid gland [2, 3]. Intra- and extracellular, “not-easily apparent” mucin is reported in thyroid carcinomas possibly in relationship with apparition of highly acidic forms of the thyroglobulin glycoprotein [3]. Thyroid adenomas or hyperplastic nodules may show this change rarely. To mention would be that thyroid nodules with prominent or extensive mucin, mainly in the stroma are reported, however as rare morphological variants [4–6]. The present cases are particular by the presence of “lake”-type zones of stromal mucin together with focal, subepithelial foci (Fig. 1). Whether indomethacin, frequently prescribed drug, may favorize or initiate such changes in the context of hyperthyroidism as would be the situation of the 3 present cases, is difficult to precise. To mention would be that in the reported indomethacin-induced gastric ulcer Wistar rats models, thyroid hormones increased the expression of both neural and acidic mucins [1]. To note would also be that in the cases of human thyroid hyperplastic nodules we have analysed, this change was of limited clinical relevance. However, detection of mucus on thyroid fine needle aspiration cytology specimens may rise the question not only of a thyroid carcinoma (primitive or secondary) but also of benign nodules [7].

In conclusion, thyroid mucin may occur in human thyroid hyperplastic nodules. Whether this change is related to indomethacin treatment in the context of hyperthyroidism, or not remains to be further investigated.
